# Evaluation of pain perception during orthodontic debonding of metallic brackets with four different techniques

**DOI:** 10.1590/1678-7757-2018-0003

**Published:** 2019-01-07

**Authors:** Delal Dara Kilinç, Gulsilay Sayar

**Affiliations:** 1 Istanbul Medipol University, School of Dentistry, Department of Orthodontics, Istanbul, Turkey

**Keywords:** Pain, Orthodontic brackets, Dental debonding, Occlusal force, Bite force

## Abstract

**Objective:**

The aim of this study was to evaluate patients’ pain levels during four different debonding procedures. The null hypothesis was that the pain perception of the patients undergoing four different debonding applications was not statistically significant different.

**Material and Methods:**

One hundred and twenty orthodontic patients who underwent orthodontic debonding were included in this study. The patients were randomly divided into 4 groups according to technique used in the patients. Debonding groups were as follows: Group 1) Conventional debonding group, Group 2) Medication group (acetaminophen was given 1 hour before debonding), Group 3) Soft bite wax group, and Group 4) Soft acrylic bite wafer group. The patients’ levels of anxiety and fear of pain were evaluated before debonding, and Numerical Rating Scale (NRS) was applied to evaluate their pain perception during debonding. Mann-Whitney U and Kruskal–Wallis tests were used to evaluate non-normally distributed data. Categorical data analysis were carried by chi-square and McNemar tests. The significance level was set at p<0.05.

**Results:**

Anxiety scores of the patients were not statistically significant between both genders and debonding groups. In the quadrants in which the patients were perceived, the highest pain level was in the left side of the mandible. The teeth in which the highest pain level was perceived were the lower left and upper right lateral incisors. Although there was no statistically significant difference among the pain scores of the patients in each group, quadrant scores of female patients showed significant differences, being the lowest scores in the soft bite wax group.

**Conclusions:**

Majority of the patients had no fear of pain before debonding. Pain levels of the patients in the conventional debonding group were not significantly different from those of the other groups, except quadrant scores of females in the soft bite wax group. The null hypothesis was accepted.

## Introduction

Orthodontic treatment procedures such as separator placement, orthodontic force application, archwire placement and activation, and debonding procedure usually involve pain and discomfort, and 90% to 95% of patients reported having pain during orthodontic treatment.^1^ ^-^ ^6^ It has been generally accepted that pain perception may be related to age, individual pain threshold, motivation, psychological condition, previous negative dental experience, and the magnitude of orthodontic force. Some previous reports showed women reported more pain experience than men,^7^ ^,^ ^8^ while other reports showed no gender differences regarding pain perception.^5^ ^,^ ^9^ ^-^ ^11^

Pain may arise during the active phases of orthodontic treatment and during the debonding procedure.^1^ ^,^ ^2^ To lessen or prevent the pain during debonding are as important as preventing enamel damage and, thus, the use of different orthodontic instruments, ultrasound, laser application, thermal heating the orthodontic adhesives, or biting occlusal bite wafers at debonding have been discussed in previous reports.^11^ ^-^ ^13^

Debonding procedure should be harmless, painless and quick.^14^ Pain and discomfort resulting from fixed orthodontic appliances, such as elastomeric separator and arch wire placement, were evaluated in previous studies,^5^ ^,^ ^6^ ^,^ ^15^ but pain perception in debonding procedure is still a poorly documented issue in orthodontics. The aim of this study was to evaluate the pain levels in different debonding applications and the patients’ anxiety levels before the procedure to determine the best debonding technique. The null hypothesis was that the pain perception of the patients undergoing four different debonding applications is not statistically significant different (conventional debonding, debonding with acetaminophen administration, debonding while biting a soft plastic wafer, and debonding with biting wax).

## Material and methods

The sample size was determined using a computer program (Minitab version 17, Minitab Inc, State Collage, Pennsylvania, USA). The calculation was made based on a significance level of 0.05 and a power of 90% to detect a clinically meaningful difference of 1 cm in NRS. For acute and traumatic pains, minimum mean change of 13 mm (median of 11 mm) was accepted as clinically significant level in visual analog pain scale.^13^ Based on this knowledge, this prospective study was carried out on 120 orthodontic patients (84 females and 36 males) at orthodontic debonding stage. This means that 2880 teeth will be included to this study. The same researcher (G.S) treated all the patients. Ethics committee of Istanbul Medipol University approved this study with the approval number 10840098-604.01.01-E.25319. An informed consent was obtained from all the patients or their parents. The inclusion criteria for this study were as follows: patients aged 12-18 years, presence of all permanent teeth except the third molars, use of upper and lower fixed orthodontic appliances (0.018 inch metallic Gemini Series Brackets −3M Unitek, Monrovia, California, USA), 0.017×0.025 inch stainless steel archwires (3M Unitek, Monrovia Calif), and bonding procedure carried out by using Transbond XT primer+Transbond XT Adhesive paste (3M Unitek, Monrovia, California, USA). In addition to these criteria, the patients were asked about having no medical problem, no medication, no dental or periodontal problem, and no craniofacial disorder. The mean age of the patients was 15.10±1.83 years at the debonding appointment.

Patients arriving at debonding appointment were enrolled to 4 different groups (n=30) determined by debonding method. The first 30 patients whose active orthodontic treatments terminated were enrolled to Group 1, the second 30 patients to Group 2, and so on, without considering the age, gender, malocclusion type, and treatment duration.

**Figure 1:**
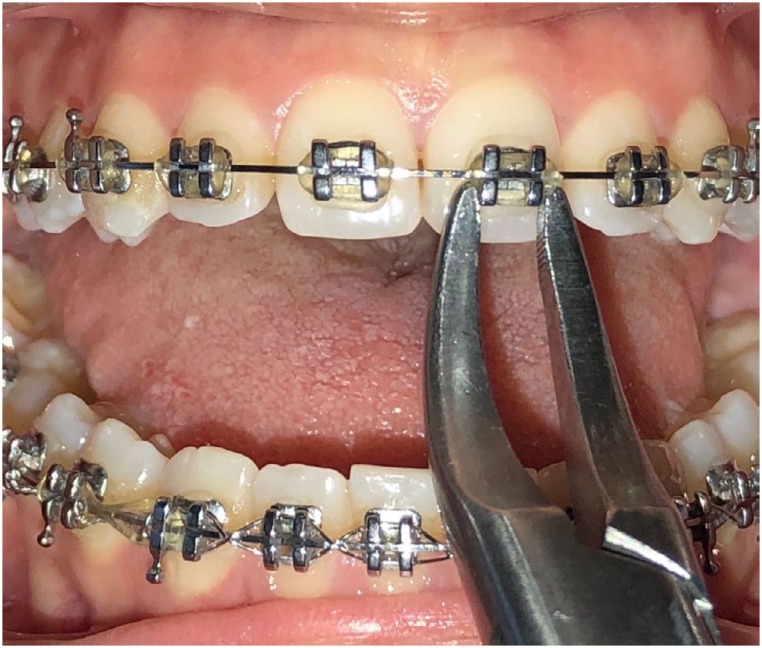
Intraoral photograph of conventional debonding

The debonding procedures applied to each group were as follows:

Group 1) Conventional debonding group: Debonding was performed with a Weingart plier. Teeth were not in contact with their counterparts during the operation. In other words, debonding was performed with an open mouth position (Figure 1).

Group 2) Medication group: A single dose of acetaminophen (acetaminophen, 500 mg tablet) was given to the patient 1 hour before debonding, and debonding was done as explained in Group I.

**Figure 2:**
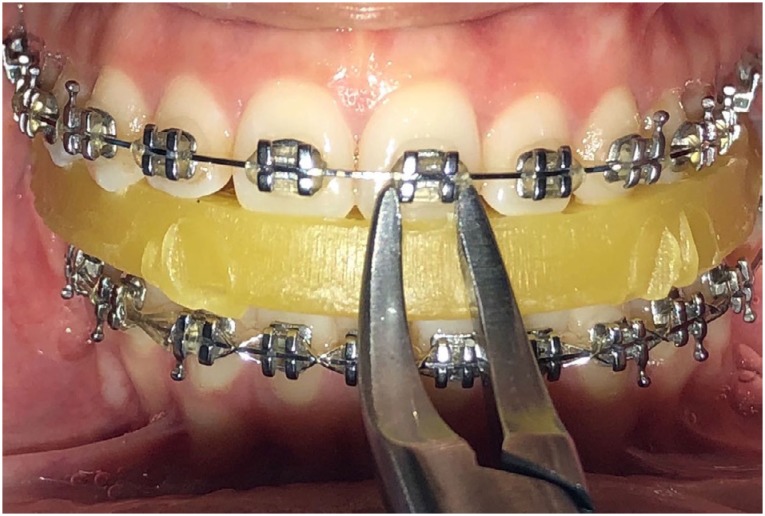
Intraoral photograph of debonding with soft bite wax

Group 3) Soft bite wax group: The patient was requested to bite on an occlusal wax (Ormco, Glendora, California, USA), and then debonding was performed with a Weingart plier (Figure 2).

**Figure 3:**
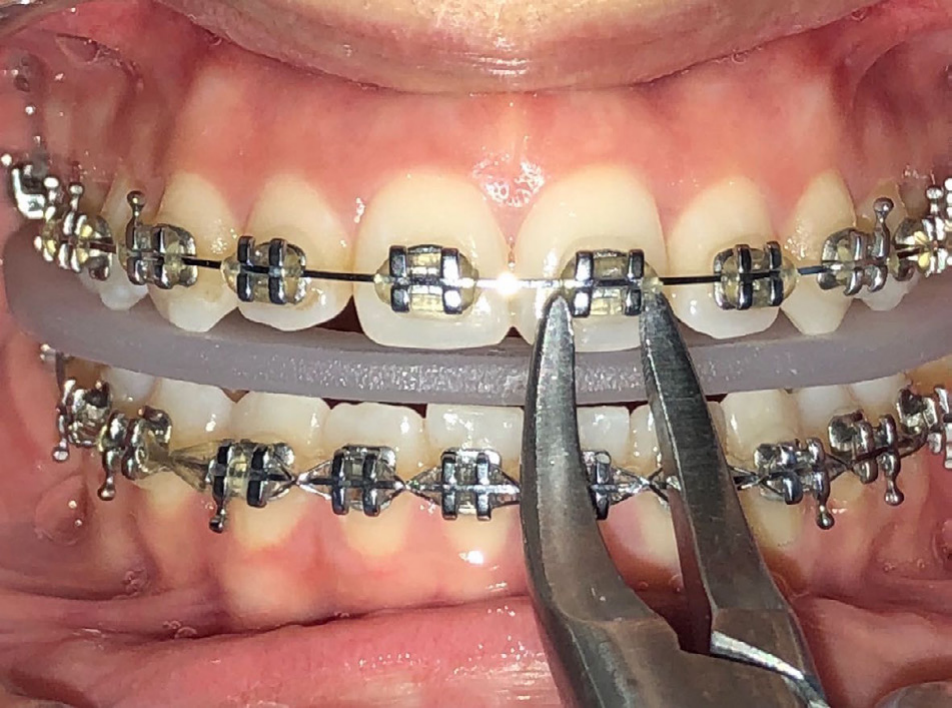
Intraoral photograph of debonding with soft acrylic bite wafer

Group 4) Soft acrylic bite wafer group: The patient was asked to bite on a soft plastic bite wafer (3M, Unitek, Monrovia, Calif), and then debonding was performed with a Weingart plier (Figure 3).

All the debondings were performed with the same Weingart pliers, beginning from the upper and lower left sides of the jaws, respectively. The Weingart plier was applied to the bracket base and squeezed the base in a mesiodistal direction. The archwires were *in situ* during debonding.

All the procedures mentioned above were managed by the same author (G.S).

Before debonding, a two-part questionnaire was applied to the patients. The dichotomous questions about the presence of anxiety and/or fear of pain were asked, and the patients answered these questions as yes or no. After debonding, numerical rating scale (NRS) was applied to evaluate the patients’ pain perception.^16^ For this purpose, the patients were asked the following questions: “in which of your teeth and in which quadrant of your jaws you had the highest pain level” and they scored the pain levels perceived on the numerical rating scale. NRS documents of each patient were numbered anonymously and number fields were masked. The other researcher (D.D.K) blinded to the groups of study assessed the NRS scores.

### Statistical analysis

Statistical analysis was performed with statistical software IBM SPSS Statistics (V23; IBM, Armonk, NY, USA). Normality of the data was evaluated with Shapiro–Wilk test. Mann-Whitney U and Kruskal-Wallis tests were used to evaluate non-normally distributed data. The analysis of categorical data was performed with chi-square test. The results were presented as median (minimum-maximum) values and interquartile ranges. The significance level was set at p<0.05.

## Results

Distribution of the patients’ anxiety scores before debonding and their between-groups comparisons and the comparisons between the genders are shown in Table 1. No between-groups differences and no gender differences in all groups regarding anxiety scores were found. Two thirds of the patients declared no fear of pain before debonding.

**Table 1:** Distribution of the anxiety scores and their between-groups and between genders comparisons

Group	Gender	No		Yes		p-value (χ^2^ between gender)
		Frequency	%	Frequency	%	
Conventional debonding	Female	12	10	8	6.6	1.000
	Male	6	5	4	3.3	
Medication	Female	12	10	14	11.6	0.467
	Male	4	3.3	_	_	
Soft bite wax	Female	16	13.3	4	3.3	1.000
	Male	6	5	4	3.3	
Soft acrylic bite wafer	Female	14	11.6	4	3.3	0.792
	Male	10	8.3	2	1.6	
Total		80	67.7	40	33.3	

Between-groups results (Kruskal-Wallis) : p=0.658 for males; p=0.292 for females

The quadrants and teeth in which the patients perceived the highest pain during debonding and their frequencies and percentages are presented in Table 2 and 3, respectively. Approximately one third of the patients (n=36) declared no pain during debonding. According to the results in Table 2, the chin area in which the frequency of pain perception was maximum (26.7%) was the lower left mandibular area.

**Table 2:** Frequencies and percentages of quadrants in which the highest pain or no pain was perceived

Quadrant	Frequency	%
Upper right	22	18.3
Lower right	18	15
Upper left	12	10
Lower left	32	26.7
No pain	36	30

**Table 3:** Frequencies and percentages of tooth numbers in which the highest pain was perceived

Quadrant	Tooth number	Frequency	%
Upper Right	11	2	2.38
	12	11	13.09
	13	2	2.38
	14	0	0,00
	15	2	2.38
	16	5	5.95
Upper Left	21	0	0,00
	22	3	3.57
	23	3	3.57
	24	0	0.00
	25	3	3.57
	26	3	3.57
Lower Left	31	8	9.52
	32	12	14.28
	33	6	7.14
	34	3	3.57
	35	0	0,00
	36	3	3.57
Lower Right	41	6	7.14
	42	2	2.38
	43	3	3.57
	44	0	0.00
	45	3	3.57
	46	4	4.76

The results of Table 3 showed the patients perceived the highest pain in different teeth, except in the upper right and left first premolar, upper left central incisor, lower left second premolar and lower right first premolar teeth. The teeth having the most pain frequency were the lower left (14.28%) and upper right (13.09%) lateral incisors.

Table 4 shows the pain scores of the patients in each group during debonding and the results of between-groups comparisons. Although there was no statistically significant difference between the groups, the patients in the soft bite wax group declared lower pain scores in both quadrant and tooth evaluations. The data in Table 4 were classified according to gender, and pain scores of the male and female patients in each group and the results of between-groups comparisons of each gender are shown in Table 5.

**Table 4:** Distribution of Numerical Rate Scale (NRS) scores regarding quadrant and teeth in which the patients perceived the highest pain and their between-groups comparisons

Groups	Quadrant Scores					Tooth Scores		
	Minimum	Maximum	Median	Interquartile Ranges	Minimum	Maximum	Median	Interquartile Ranges
Conventional debonding	0	6	6	3	3	8	6	3
Medication	1	10	6	3	0	10	6	3
Soft bite wax	0	7	3	3	1	8	3	3
Soft acrylic bite wafer	0	9	6.5	6	1	10	6.5	6
p-value	0.056				0.387			

p>0.05 Kruskal-Wallis test

**Table 5:** Distribution of Numerical Rate Scale (NRS) scores in Table 4 according to gender and their independent between-groups comparisons in female and male groups

Gender	Groups	Quadrant Scores			Tooth Scores		
		Median	Minimum	Maximum	Median	Minimum	Maximum
Female	Conventional debonding	6ab	0	8	7	3	8
	Medication	6b	1	10	6	4	10
	Soft bite wax	0.5a	0	7	3	1	8
	Soft acrylic bite wafer	0ab	0	9	7	1	10
	p-value	0.023*			0.673		
Male	Conventional debonding	5	4	7	5	5	8
	Medication	3.5	2	5	3	0	6
	Soft bite wax	0	0	3	4	3	5
	Soft acrylic bite wafer	0	0	7	6.5	6	7
	p-value	0.097			0.287		

* P<0.05 Kruskal-Wallis and Mann-Whitney U tests

There is no statistical difference between the median values that marked with the same letters (a,b,ab)

As can be seen from Table 5, quadrant scores of females showed statistically significant differences between the groups. Soft bite wax and soft acrylic bite wafer groups showed lower pain scores. These two groups also showed lower pain scores in males, although it was not statistically significant (p=0.097).

## Discussion

Bond strength is important for maintaining orthodontic treatment efficiency, but easy debonding of the brackets is preferred at the end of the treatment. Many kinds of debonding methods have been suggested to lessen the patient discomfort. These debonding methods include ultrasonic instrumentation, laser irradiation and electrothermal heating, using special pliers. In addition to these methods, modified adhesive resins containing thermoexpandable microcapsules have been used to lessen the pain and discomfort.^3^ ^,^ ^11^ ^,^ ^17^ ^-^ ^19^

Pain is an inherently subjective symptom, and thus no objective method exists for its assessment. Visual analog scale (VAS), numerical rating scale (NRS), and verbal rating scale (VRS) are commonly used measurement instruments to quantify pain intensity of the patients. The comparative studies regarding these instruments showed no statistically significant difference among them.^17^ ^,^ ^20^ In this study, numerical rating scale was used to assess the pain perceived during debonding because of its easy application.

Debonding procedures should be harmless, painless and quick.^21^ Economically acceptable and clinically easy and useful techniques are preferred in clinical applications. A complex debonding technique is not useful for the clinical perspective. For this purpose, we aimed to compare the conventional debonding technique with the modified ones. Soft bite wax and soft acrylic bite wafer were used to stabilize the teeth during debonding. A prophylactic analgesic was used to prevent pain in another group. As opposed to the procedure used in this study, Polat and Karaman^22^ (2005) used four different analgesic agents to prevent pain after bonding and archwire placement, and they compared the effects of analgesics through placebo. As a result, they concluded that acetaminophen lessened orthodontic pain more effectively than placebo.

Pain perception has been reported in different phases of the orthodontic treatment. For debonding, not pain perception but different effects of debonding procedure generally has been investigated in literature.^2^ ^,^ ^14^ ^,^ ^17^ ^,^ ^22^ ^,^ ^23^ The factors causing pain and discomfort were studied by different authors, revealing that gender, tooth type, jaw side and the tooth restorations had weak relations with discomfort. Tooth type may affect discomfort more than the other variables. Two factors may affect patient discomfort at the time of debonding: tooth mobility and direction of force application. Intrusive forces can be tolerated because the organization of periodontal structures is established to resist the intrusive forces of mastication. At debonding time, stabilization of the teeth by advising the patient to bite on a cotton roll may diminish discomfort of the patient. Stabilizing the teeth with a finger can also be helpful for minimizing discomfort.^13^ Similar forces can result in different individual responses.^24^

Williams and Bishara^12^ (1992) reported that sex, tooth type, tooth mobility, quick application of debonding force, and force direction have significant effects on the discomfort threshold in debonding. They also stated that the type of debonding instrument or bracket is not related to the pain threshold. On the other hand, Pithon, et al.^21^ (2015) investigated different debonding instruments and found debonding with a lift-off debonding instrument caused significantly lower pain levels than those carried out by the other instruments. In this study, all the patients were debonded with the same bracket removing plier to standardize the procedure. In addition, molar debondings and evaluations were included to the study protocol as opposed to the study carried out by Bavbek, et al.^25^ (2016).

It has been known that intrusive forces in debonding are tolerable force types.^12^ ^,^ 25 ^,^ ^26^ ^,^ Mangnall, et al.^26^ (2013) evaluated patients’ pain experiences during debonding with a soft acrylic wafer or conventional debonding and reported that biting a soft acrylic bite wafer could be useful to reduce pain. This study showed that there was no significant difference in pain scores of the investigated groups, although the soft bite wax group had the lowest pain scores (Table 4).

The location of the tooth has an impact on the degree of pain,^25^ being the debonding of incisors more painful than that of posterior teeth.^15^ ^,^ ^21^ This phenomenon may be related with the tactile sensory threshold, since this threshold is about 1 gram in the anterior portion of the dentition in normal subjects and gradually increases toward the posterior segment, ranging from 5 to 10 gram.^21^ According to Mangnall, et al.^26^ (2013), a greater debonding force is distributed to the *per* unit area in the lower anterior region than in the posterior, and thus greatest pain was perceived in the lower anterior teeth (39%) followed by the upper right posterior teeth (18%). The authors also stated that debonding was started from the upper right side, and thus the first debonded quadrant was remembered as more painful.^26^ In our study, debonding was made beginning from the upper left side toward the upper right posterior region, followed by the lower left quadrant around to the lower right quadrant. The highest pain level was found in the lower left quadrant. As explained by Mangnall et al.^26^ (2013), explanation of why the lower left quadrant was reported as the most painful is difficult, It may have been resulted from the torsional forces applied to the teeth during debonding.

Pre-debonding anxiety may induce pain during debonding. Pre-debonding anxiety levels of the male and female patients in each group were determined, and no significant difference between genders was observed. Between-groups, anxiety scores were also not statistically significant in both females (p=0.292) and males (p=0.658). This finding is consistent with the study by Williams and Bishara^12^ (1992), who noted that gender difference has little influence on pain. Koyama, et al.^27^ (2005) noted that the subjective pain experience is related to expectations of pain and alters the brain mechanism, in other words, positive expectations result in a reduced pain experience.

A statistically significant difference was observed in the quadrant scores of female patients. Soft bite wax group showed lower debonding pain levels than the other groups and no significant differences among the other groups was observed. The subjectivity of pain perception shown in this study was similar to that in the previous reports.^25^ ^,^ ^28^

It might be thought that this study had some limitations. For example, there may have been a bias in patient recruitment into the different groups because this study is a controlled clinical trial. Again, adding the patients with ceramic brackets could enhance the scientific value of the study.

## Conclusions

The results of this study can be summarized as follows:

1. Pre-anxiety scores of the patients showed no difference between genders and groups.
2. The quadrants and teeth in which the patients perceived the highest pain level was the left side of mandible and lower left and the upper right lateral incisors, respectively.
3. No significant difference among the four different debonding techniques was found. The pain level perceived in conventional debonding technique was not statitically different from the others.
